# Beyond Zoonoses in One Health: Non-communicable Diseases Across the Animal Kingdom

**DOI:** 10.3389/fpubh.2021.807186

**Published:** 2022-01-26

**Authors:** B. Natterson-Horowitz, Marion Desmarchelier, Andrea Sylvia Winkler, Hélène Carabin

**Affiliations:** ^1^Division of Cardiology, David Geffen School of Medicine at University of California, Los Angeles (UCLA), Los Angeles, CA, United States; ^2^Department of Human Evolutionary Biology, Harvard University, Boston, MA, United States; ^3^Department of Clinical Sciences, Faculté de Médecine Vétérinaire, Université de Montréal, Saint-Hyacinthe, QC, Canada; ^4^Department of Neurology, Center for Global Health, Technical University of Munich, Munich, Germany; ^5^Centre for Global Health, Institute of Health and Society, University of Oslo, Oslo, Norway; ^6^Department of Pathology and Microbiology, Faculté de Médecine Vétérinaire, Université de Montréal, Saint-Hyacinthe, QC, Canada; ^7^Department of Social and Preventive Medicine, École de Santé Publique (ESPUM), Université de Montréal, Montréal, QC, Canada; ^8^Groupe de Recherche en Épidémiologie des Zoonoses et Santé Publique (GREZOSP), Saint-Hyacinthe, QC, Canada; ^9^Centre de Recherche en Santé Publique (CReSP), Montréal, QC, Canada

**Keywords:** non-communicable disease (NCD), one health, public health, environmental exposure, animal surveillance

## Introduction

**Figure 1:**
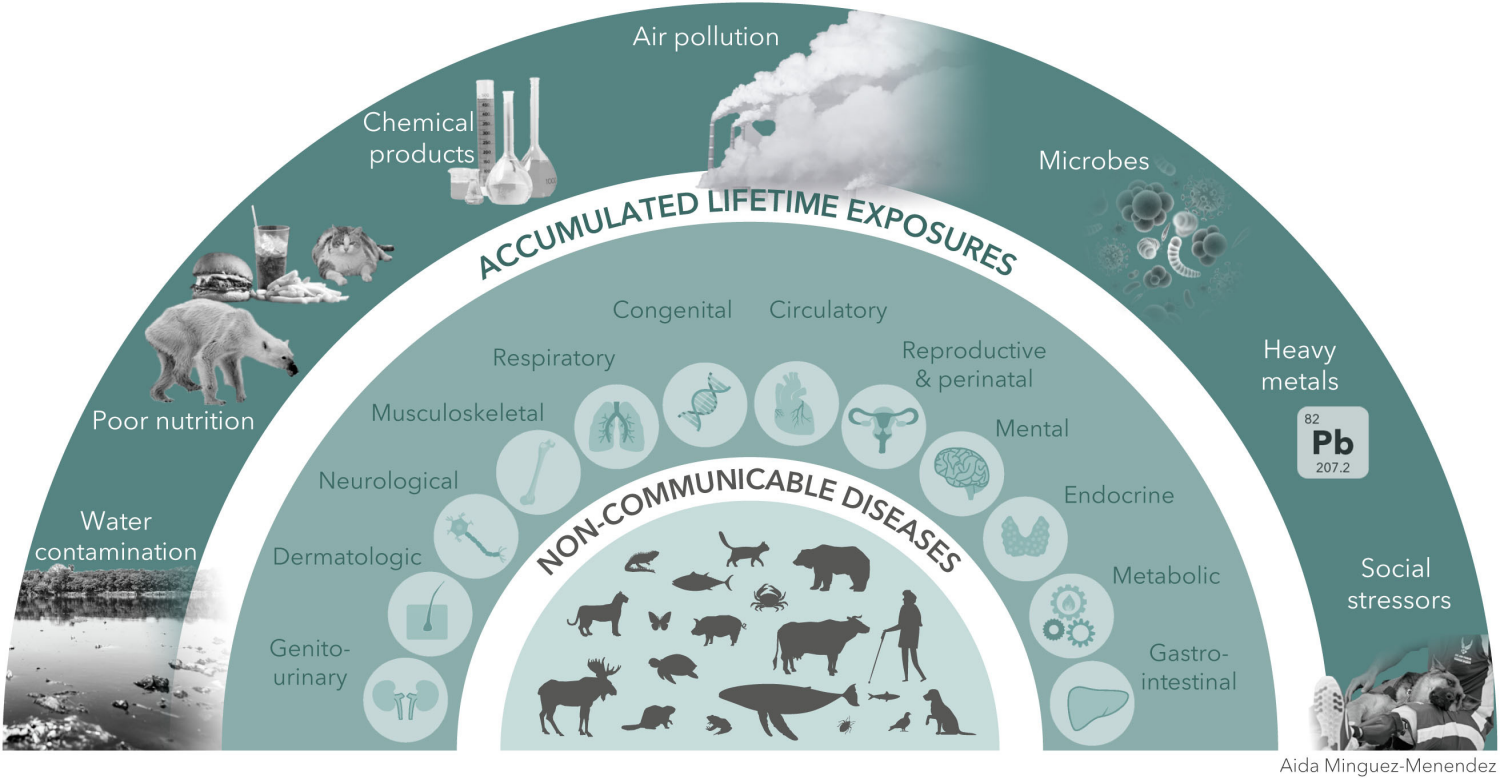
Shared environmental exposures contribute to NCDs across species.

Greater than 70% of all human deaths are due to non-communicable diseases (NCDs) (1). Increasingly environmental exposures, from air pollution, second-hand smoke, heavy metals, phthalates, pesticides, and endocrine disrupting chemicals, have been linked to a range of NCDs including many cancers, cardiopulmonary diseases, congenital abnormalities and other pathologies (2). Many of the same environmental effects that elevate disease risk in humans, also do so in other species. In fact, the smaller size of many non-human animals, shorter lifespans and their greater exposure to environmental hazards may accelerate the natural history of pathology (2). Between domestication, urbanization and increased land use, the line demarcating “human” and “animal” environments is increasingly blurred and health professionals have an opportunity to turn to animal models to address human health. For instance, surveillance for NCDs in other species has the potential to alert human health professionals to environmental hazards before the emergence of pathology in human populations (2). The importance of animal sentinels in detecting and combating communicable diseases has been made strikingly clear over the past decades (3) as well as by the recent COVID-19 pandemic. The high percentage of communicable diseases with zoonotic origins reveals the need for broad surveillance of wild and domestic animal populations that may carry pathogens that can infect in humans (4). However, despite increased awareness of and resources for surveillance of animal sentinels with zoonotic diseases, far fewer resources have been directed toward surveillance of animal populations for NCDs (5) and widespread reforms addressing the connection between human, animal and environmental health remain elusive. Here, we briefly present examples of NCDs in non-human animals that derive from shared environmental sources to emphasize the utility and need for species-spanning NCD surveillance (Figure 1).

## NCDS, Environments, and Threats

Animal sentinels can provide critical information on a number of high impact human NCDs including cancers, developmental abnormalities and neuropsychiatric disorders. For example, companion animals exposed to second-hand smoke are at increased risk for lung cancer and household chemicals cause bladder and other cancers in both dogs and humans living in the same households (6). Strong evidence now links various carcinomas to polycyclic aromatic hydrocarbons (PAH), persistent organo-halogen compounds (POCs) and other endocrine disrupting agents in free-living marine mammals, rodents, and some bird species (7–9). Due to their heightened sensitivity, ferrets and birds have also been efficient sentinels for airborne toxic particles (10, 11), which can lead to lung cancer. Surveillance of animal populations can provide human health professionals with early warnings of environmental carcinogens.

Environmental factors, especially exposure to fine particulate matter, have been implicated in over 25% of ischemic heart disease related deaths (12). Atherosclerosis has been identified in the vasculature of a wide range of animals in wild, captive, companion and agricultural settings great apes (13), parrots (14, 15), domestic cats (16), swine, and horses (17). The vulnerability of other animals to atherosclerosis and the increasingly strong linkage between environmental factors and atherogenesis point to a major underleveraged source of insights. Surveillance of animal populations for atheroma may expose heightened presence of atherogenic environmental exposures, such as particulate matter.

A growing list of environmental contaminants are now recognized to increase risk of congenital syndromes in humans (18). These include: plastic and related chemicals that delay fetal brain development, endocrine disrupting chemicals and altered sex skew, genitourinary abnormalities, and a host of other disorders. Such endocrine disruptors are found in a wide range of everyday products, including cosmetics, toys, plastic containers and food (19). In the wild, environmental contaminants contribute to declining numbers of bees due to neonicotinoid-induced impaired social behavior (20), amphibians going through mass extinction due to multiple environmental disturbances (21), and feminized male freshwater fish due to endocrine disrupting compounds (22), which all have direct relevance to human health. Surveillance for congenital disorders in animals may expose otherwise undetected links between environmental toxins and congenital abnormalities in humans.

In humans, NCDs are associated with social determinants of health and have been linked to social and environmental factors such as poverty, gender, race, food insecurity or being exposed to violence (1, 23–25). Lifestyles contribute to NCDs in both animals and humans. For example, diet and inactivity which are linked to cardiovascular disease in humans are also believed to increase risk in captive and companion birds (14). Obesity—-and its associated comorbidities—-has also been linked to diet and levels of activity in humans, cats, dogs, and other species (26–28). In some cases, the human-animal bond can be leveraged to motivate lifestyle behavioral changes with positive health effects (29).

The presence of an abusive individual in a household has been shown to increase the risk faced by all individuals, regardless of species-type, living with the abuser (2, 11, 30–32). While the effects on domestic animals have not been rigorously studied, a history of abuse, neglect, or prior mismanagement is reported as a major co-occurrent factor in human dog bite-related fatalities (30). Developmental vulnerability to early trauma has been linked to increased risk of mental illness in later life in humans (33, 34) and a growing body of research supports similar relationships in dogs (32, 35). Since animal abuse often precedes abuse of humans, it has been suggested that pets can be sentinels for potential violence in human homes and this connection points to a novel approach to reducing risk of trauma and subsequent psychopathology in humans.

Mental illness affects 15–20% of humans (32, 33) and environmental factors are increasingly implicated in a wide range of neuropsychiatric disorders. As estimates of humans affected by mental illness rise, the relevance of neuropsychiatric health of other species has increasing relevance to human mental health. In Minamata, Japan, for example a mysterious neuropsychiatric disorder in humans was not identified as methylmercury poisoning until cats in the area began behaving aberrantly (36). Canine and feline anxiety and compulsive disorders share features with human correlates (37) and feather-damaging behavior in parrots is a model for trichotillomania (38, 39). Unfortunately, there has been limited knowledge exchange between human psychiatry and veterinary behavioral medicine, which was only recognized as a specialty of veterinary medicine in 1993. Investigating common processes of brain dysfunction and their management strategies across species, such as post-traumatic stress disorder (PTSD) in military canines and humans, might be valuable in the fight against mental illness stigma, which is recognized as an important barrier to care. This has particular salience for health professionals, who have higher rates of depression than the general public, with veterinarians suffering the highest rate of suicide of all (40).

## Discussion

In 1999, the Bronx Zoo's veterinary pathologist had concerns that a parallel epidemic of encephalitis among elderly people might be related to her recent avian die off. Her efforts to engage the CDC and the New York State Health department in an investigation were at first met with resistance, thus delaying the identification of West Nile Virus as the common pathogen. Since that time, some progress has been made with the creation of a few joint surveillance systems for infectious agents and antimicrobial resistance. However, perhaps as a consequence of a long tradition of human exceptionalism in medicine, many physicians still lack awareness of the connection between NCDs in humans and other species. In fact, traditional medical education has a poor record of teaching the similarities between human and animal health and has largely ignored the connection between human and wildlife health (41). As NCDs continue to surge in human communities and as the connection between these diseases and the environment grows more clear, the health of animals sharing these vulnerabilities is of great importance.

While strengthened evidence for the role of environments in NCD-related human deaths has generated appropriate concern among human health professionals, the low level of resources directed at surveillance efforts indicates that the importance of animal sentinels appears to be not well-understood. In a recently published systematic review of animal surveillance, most studies focused on zoonotic and infectious pathologies rather than NCDs (5). Additionally, numerous barriers were identified which limit effective surveillance efforts including problems with data standardization, collection and sharing, poor integration of effort across disciplines, and under engagement by private sector stakeholders. As a consequence, the authors report “the systems cannot meet their objective, such as the detection of health events in animals to prevent human cases or the attribution of sources for human cases” remains underdeveloped (5).

While the lessons of West Nile and other zoonotic diseases helped increase collaboration across the fields of human and veterinary medicine, it is time to apply these lessons from zoonoses to non-communicable health challenges threatening humans, animals and their shared environments around the globe. The opportunity is tremendous, yet it remains unrealized. Employing One Health frameworks can provide innovative solutions to prevent and treat NCDs across the animal kingdom. Increased awareness among human health professionals and stakeholders of the shared vulnerability across species of environmentally- linked NCDs should ignite interest and ideally mobilize resources for NCD surveillance across species (42). These efforts could not only improve the health of humans, but of all species sharing exposures in increasingly hazardous environments.

## Author Contributions

BN-H consolidated the final version of the manuscript. All authors contributed to idea conception, draft manuscript preparation, reviewed the results, and approved the final version of the manuscript.

## Funding

HC was supported in part by the Canada Research Chair in Epidemiology and One Health (Grant no. CRC 950-231857).

## Conflict of Interest

The authors declare that the research was conducted in the absence of any commercial or financial relationships that could be construed as a potential conflict of interest.

## Publisher's Note

All claims expressed in this article are solely those of the authors and do not necessarily represent those of their affiliated organizations, or those of the publisher, the editors and the reviewers. Any product that may be evaluated in this article, or claim that may be made by its manufacturer, is not guaranteed or endorsed by the publisher.
